# Novel treatment option for MUC16-positive malignancies with the targeted TRAIL-based fusion protein Meso-TR3

**DOI:** 10.1186/1471-2407-14-35

**Published:** 2014-01-21

**Authors:** Gunjal Garg, Jesse Gibbs, Brian Belt, Matthew A Powell, David G Mutch, Peter Goedegebuure, Lynne Collins, David Piwnica-Worms, William G Hawkins, Dirk Spitzer

**Affiliations:** 1 Department of Obstetrics and Gynecologic Oncology, Washington University School of Medicine, St. Louis, MO 63110, USA; 2 Department of Surgery, Washington University School of Medicine, St. Louis, MO 63110, USA; 3 Siteman Cancer Center, Washington University School of Medicine, St. Louis, MO 63110, USA; 4 Molecular Imaging Center, Mallinckrodt Institute of Radiology, BRIGHT Institute, Departments of Cell Biology & Physiology, and Developmental Biology, Washington University School of Medicine, St. Louis, MO 63110, USA; 5 Current address: Cancer Systems Imaging Department, Division of Diagnostic Imaging, The University of Texas M.D. Anderson Cancer Center, T. Boone Pickens Academic Tower, 1400 Pressler Street, Unit 1479, Houston, TX 77030, USA; 6 Department of Surgery, Washington University School of Medicine, 660 S. Euclid Avenue, Campus Box 8109, St. Louis, MO 63110, USA

**Keywords:** TRAIL, Mesothelin, MUC16, Biomarker, Fusion protein, Meso-TR3

## Abstract

**Background:**

The targeted delivery of cancer therapeutics represents an ongoing challenge in the field of drug development. TRAIL is a promising cancer drug but its activity profile could benefit from a cancer-selective delivery mechanism, which would reduce potential side effects and increase treatment efficiencies. We recently developed the novel TRAIL-based drug platform TR3, a genetically fused trimer with the capacity for further molecular modifications such as the addition of tumor-directed targeting moieties. MUC16 (CA125) is a well characterized biomarker in several human malignancies including ovarian, pancreatic and breast cancer. Mesothelin is known to interact with MUC16 with high affinity. In order to deliver TR3 selectively to MUC16-expressing cancers, we investigated the possibility of targeted TR3 delivery employing the high affinity mesothelin/MUC16 ligand/receptor interaction.

**Methods:**

Using genetic engineering, we designed the novel cancer drug Meso-TR3, a fusion protein between native mesothelin and TR3. The recombinant proteins were produced with mammalian HEK293T cells. Meso-TR3 was characterized for binding selectivity and killing efficacy against MUC16-positive cancer cells and controls that lack MUC16 expression. Drug efficacy experiments were performed in vitro and in vivo employing an intraperitoneal xenograft mouse model of ovarian cancer.

**Results:**

Similar to soluble mesothelin itself, the strong MUC16 binding property was retained in the Meso-TR3 fusion protein. The high affinity ligand/receptor interaction was associated with a selective accumulation of the cancer drug on MUC16-expressing cancer targets and directly correlated with increased killing activity in vitro and in a xenograft mouse model of ovarian cancer. The relevance of the mesothelin/MUC16 interaction for attaching Meso-TR3 to the cancer cells was verified by competitive blocking experiments using soluble mesothelin. Mechanistic studies using soluble DR5-Fc and caspase blocking assays confirmed engagement of the extrinsic death receptor pathway. Compared to non-targeted TR3, Meso-TR3 displayed a much reduced killing potency on cells that lack MUC16.

**Conclusions:**

Soluble Meso-TR3 targets the cancer biomarker MUC16 in vitro and in vivo. Following attachment to the tumor via surface bound MUC16, Meso-TR3 acquires full activation with superior killing profiles compared to non-targeted TR3, while its bioactivity is substantially reduced on cells that lack the tumor marker. This prodrug phenomenon represents a highly desirable property because it has the potential to enhance cancer killing with fewer side-effects than non-targeted TRAIL-based therapeutics. Thus, further exploration of this novel fusion protein is warranted as a possible therapeutic for patients with MUC16-positive malignancies.

## Background

TRAIL (TNF-related apoptosis-inducing ligand) is a member of the TNF (Tumor Necrosis Factor) superfamily and induces apoptosis upon binding to its death receptors DR4 and DR5 [1-3]. Two unique properties have been described for TRAIL that renders this cytokine highly attractive for cancer therapy. First, it selectively acts on malignant/transformed cells despite the fact that many normal host cells also express the activating death receptors [4,5]. This cancer selective advantage can be lost under inflammatory conditions. For example, hepatocytes under the influence of chemotherapy can become susceptible to TRAIL due to upregulation of activating death receptors [6-8]. Secondly, TRAIL induces the death receptor pathway independent of p53, a tumor suppressor which plays an important role in tumor formation and progression [9].

To further optimize the treatment efficiency of TRAIL-based cancer drugs, targeting concepts have been evaluated which serve two main purposes: 1) to accumulate the drugs at the tumor site and thereby limiting sequestration by non-transformed host cells (reduced side-effects) and 2) to immobilize TRAIL to the cancer cell membrane which converts a soluble drug into a membrane-bound analog. This conversion results in enhanced death receptor signaling and cell death induction [10]. Various approaches have been described for targeting death receptor ligands to the tumors including construction of TRAIL fusion proteins using antibody fragments directed against overexpressed tumor antigens [11].

We recently described a method of producing soluble TRAIL from mammalian cells by genetic fusion [12]. This novel human TRAIL trimer, designated TR3, is comprised of three consecutive extracellular TRAIL domains fused together in a head-to-tail configuration. It is devoid of artificial linker sequences and is therefore “all-human”. The killing activity of TR3 is comparable to that of commercially available recombinant TRAIL (rTRAIL). TR3 is more stable at physiologic temperatures and has a superior pharmacologic profile. Furthermore, genetic modification of the TR3 drug platform is feasible allowing delivery of preassembled bioactive TRAIL trimers to selected cell surface markers in a stoichiometrically-controlled fashion [12].

In our current study, we sought to deliver TR3 selectively to the cancer cells employing a native, high-affinity ligand/receptor interaction between mesothelin and MUC16, also known as CA125 [13]. MUC16 is an established biomarker in ovarian cancer that is also overexpressed in other malignancies such as pancreatic [14,15] and breast cancers [16]. We thus generated a mesothelin-TR3 fusion protein, designated Meso-TR3, and performed biochemical and functional characterization experiments *in vitro* and *in vivo*. One of the key characteristics of this novel cancer drug is its potency on MUC16-positive cancer cells with reduced killing abilities on MUC16-negative cancer cells. This “prodrug” phenomenon is highly desirable as it is predicted to substantially reduce potential off-target toxicities of an otherwise highly effective cancer therapeutic.

## Methods

### Cells and reagents

All cell lines used in the experiments were obtained from the American Type Culture Collection (ATCC, Manassas, VA). Recombinant human TRAIL was purchased from Enzo Life Sciences (formerly BIOMOL, International, Farmingdale, NY). Mouse anti-mesothelin mAb (clone K1) was purchased from Santa Cruz Biotech (Santa Cruz, CA).

### Construction of plasmids and protein production

Soluble mesothelin was generated by inserting a FLAG tagged, GPI anchor-deleted mature form of a mesothelin cDNA into the expression plasmid sT-DAF [17] that contains a signal peptide to ensure protein secretion from mammalian cells. The FLAG tag was inserted to distinguish genetically engineered mesothelin from the endogenously expressed mesothelin on ovarian cancer cells. The basic TR3 expression plasmid was described previously [12], modified to include an internal 6 × His tag. Meso-TR3 was generated by N-terminal insertion of soluble mesothelin into the TR3 drug platform. The recombinant TR3 forms, soluble mesothelin, and DR5-Fc were produced by transient expression in HEK293T cells using Gibco Opti-Mem serum free medium and TransIT-293 transfection reagent (MIR2700, MIRUS Bio LLC, Madison, WI), as per the manufacturer’s instructions. To obtain concentrated protein stocks, the supernatants were applied to centrifugal filter devices with a 10 kDa molecular cut-off (Centricon Plus-20, Millipore, Billerica, MA). DR5-Fc was purified using Protein A columns as per the manufacturer’s instructions (Pierce, Rockford, IL).

### Flow cytometry

To assess the MUC16 surface expression, cells were incubated with anti-MUC16 mAb X75 (Abcam, Cambridge, MA) followed by staining with FITC conjugated secondary antibody (anti-mouse IgG, Sigma-Aldrich, St. Louis, MO). For binding studies of Meso-TR3 to OVCAR3 cells, Meso-TR3 was first preincubated with DR5-Fc. Under constant agitation, OVCAR3 cells were then combined with the Meso-TR3/DR5-Fc complexes for one hour, washed and subsequently incubated with anti-FLAG mAb M2 (Sigma-Aldrich, St. Louis, MO), followed by FITC conjugated secondary Ab (anti-mouse IgG, Sigma-Aldrich) and analyzed employing flow cytometry (FACSCalibur, BD Biosciences, San Jose, CA).

### Confocal microscopy

OVCAR3 and HeLa cells were cultured for 24 h on millicell EZ slides (Millipore) and then fixed with 4% paraformaldehyde. The cells were blocked with serum-free Protein Block (Dako, Carpinteria, CA). Primary antibodies for FLAG (mouse mAb M2) and MUC16 (rabbit pAb, Sigma-Aldrich) were allowed to bind for 2 h, washed and detected with the respective secondary Abs Alexa Fluor 488 goat anti-mouse IgG (Invitrogen, Carlsbad, CA) and Alexa Fluor 555 goat anti-rabbit IgG (Invitrogen). Confocal images were taken on a Zeiss LSM 510 META Confocal Laser Scanning Microscope (Zeiss, Jena, Germany).

### Analysis of cell death

OVCAR3, HeLa, and Jurkat cells were seeded in 96 well plates. Unless otherwise stated, the cells were treated the following day and assayed 18 hours later using CellTiter-Glo Luminiscent Viability Assay (Promega, Madison, WI). Data were recorded with a SpectraMax Gemini microplate spectrofluorometer, Molecular Devices (Silicon Valley, CA).

### Immunoblotting

Transfection supernatants were submitted to 10% SDS-PAGE and transferred onto a nitrocellulose membrane. After blocking with dry milk, the membranes were incubated with a rabbit primary antibody (anti-human TRAIL pAb, Peprotech, Rocky Hill, NJ) followed by an HRP-conjugated secondary antibody (goat anti-rabbit IgG, Santa Cruz Biotech) and developed with Immunstar Western C kit (Bio-Rad, Hercules, CA) using the Chemidoc XRS plus Imaging system (Bio-Rad).

### Animals

Eight week old female Nonobese Diabetic/Severe Combined Immunodeficiency mice (NOD/SCID; Harlan, IN) were used as hosts for tumor xenografts. Human OVCAR3 tumor cells (2 × 10^6^ cells per animal), stably expressing the eYFP-Luciferase fusion protein were injected into the abdomen (i.p.) prior to drug treatments (300 μL/mouse/day). Procedures involving mice were approved by the Washington University Animal Studies Committee and conducted in accordance with the guidelines for the care and use of laboratory research animals established by the NIH.

### Bioluminescence imaging

For bioluminescence imaging of living animals, mice were injected intraperitoneally with 150 μg/g D-luciferin (Biosynth, Naperville, IL) in PBS, anesthetized with 2.5% isoflurane, and imaged with a charge-coupled device (CCD) camera-based bioluminescence imaging system (IVIS 100; Caliper, Hopkinton, MA) with exposure times ranging from 1 to 60 seconds as described elsewhere [18]. The signals were displayed as photons/sec/cm^2^/sr.

### Statistical analyses

Treatment efficiency of in vitro killing assays are presented as means ± SEM. Treatment efficiency in vivo is expressed as reduction in bioluminescence signal relative to the individual animal’s baseline intensity as means ± SEM. Statistical significance is defined as P < 0.05 and was calculated employing analysis of variance (one-way ANOVA, Tukey’s Multiple Comparison Test) and the Student’s t-test (unpaired) as indicated using GraphPad Prism (V 4.02) software.

## Results

### Design, generation and biochemical characterization of Meso-TR3

**Figure 1:**
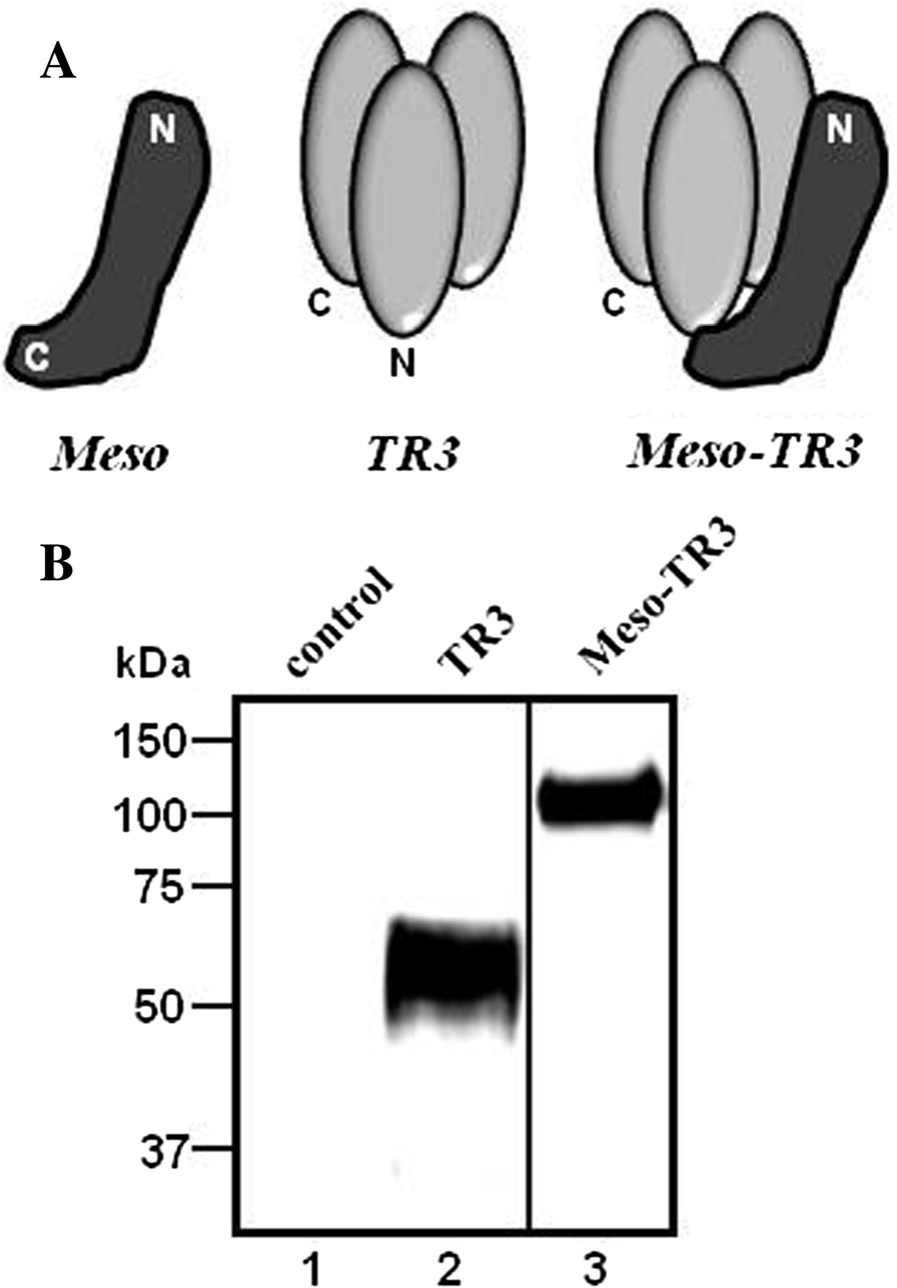
**Design and biochemical characterization of the MUC16-targeted TRAIL trimer TR3. A**, Schematic representation of the proteins used in this study. Soluble mesothelin (Meso) containing an N-terminal FLAG tag (not shown), the parental TRAIL drug platform TR3 (center) and the MUC16-targeted mesothelin-TR3 fusion protein (Meso-TR3) were produced by transient transfection of HEK293T cells. N, amino-terminus; C, carboxyl-terminus. **B**, Western blot analysis (reducing conditions) documents the molecular weights of TR3 (≈60 kDa, lane 2) and Meso-TR3 (≈100 kDa, lane 3) using anti-TRAIL pAb. Supernatant from mock-transfected HEK293T cells served as a negative control (lane 1).

Soluble mesothelin has been shown to bind to MUC16 rapidly and with high affinity [13]. Since endogenous mesothelin is attached to the cell surface via a GPI anchor [19,20], we designed a secreted form of the glycoprotein by deleting its GPI signal sequence (Figure 1A, Meso). For immunologic detection purposes, we included a FLAG epitope tag, located at the amino-terminus of the secreted protein (not shown). The recombinant protein was produced in HEK293T cells and Western blot analysis confirmed its identity with a molecular weight of ≈ 40 kDa (not shown). To convert TR3 (Figure 1A, center) into a MUC16-targeted cancer drug, we inserted the entire cDNA of soluble mesothelin (including the N-terminal FLAG tag) to the 5′-terminus of a TR3 expression plasmid (Figure 1A, Meso-TR3). The resulting genetic constructs were expressed in mammalian 293T cells and characterized by Western blot analysis. Meso-TR3 was identified as a fusion protein with an apparent molecular weight of ≈ 100 kDa, with the parental molecule TR3 being ≈ 60 kDa (Figure 1B), consistent with a size reduction of 40 kDa, the molecular weight of the mature form of soluble human mesothelin. Using this method of molecular weight detection, we were unable to identify fragmentation products, suggesting lack of proteolytic degradation of Meso-TR3.

### Meso-TR3 binds to MUC16 on the cancer cell membrane

**Figure 2:**
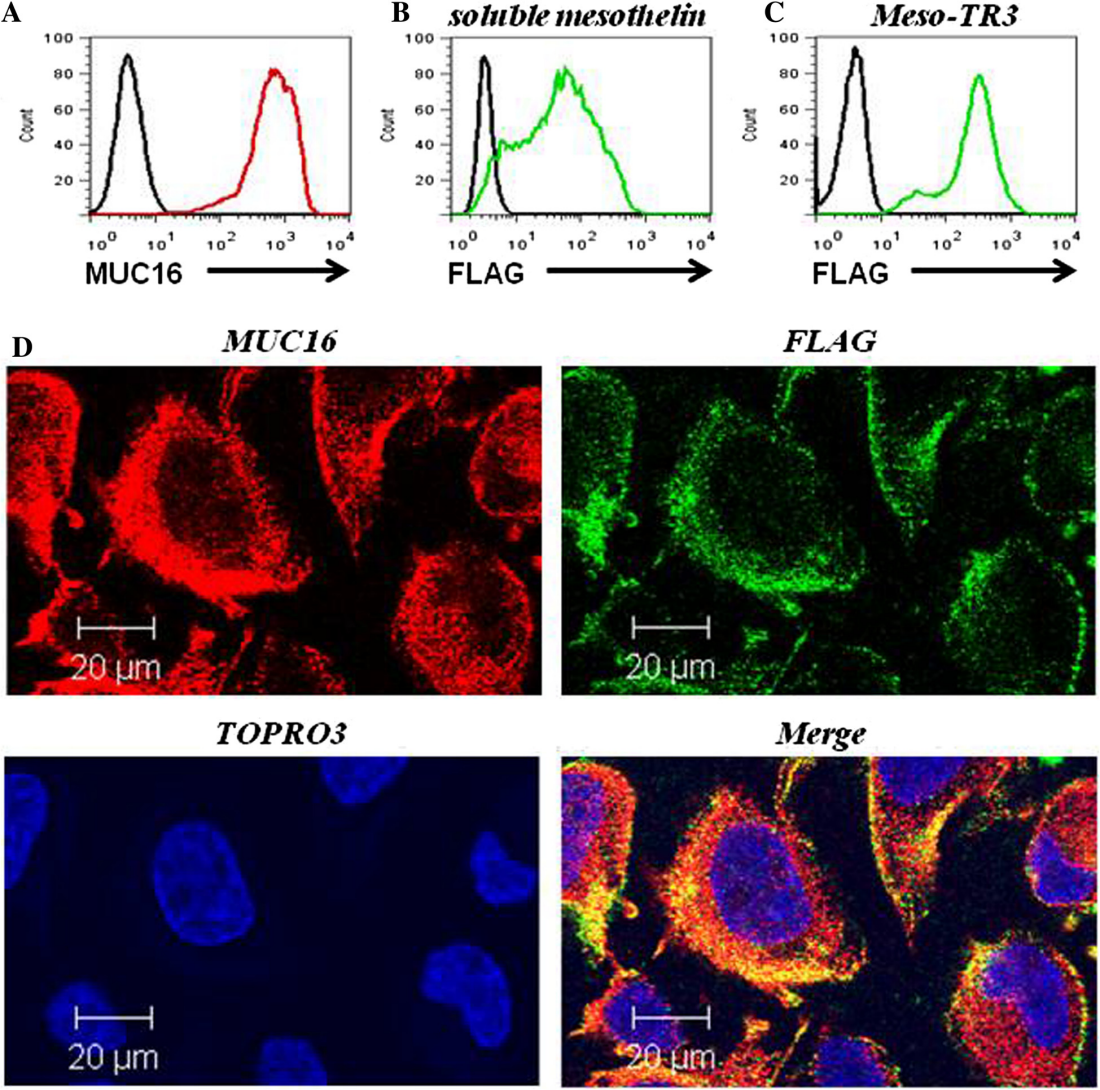
**Meso-TR3 binding to MUC16-expressing cancer targets. A**, FACS-analysis of OVCAR3 cells assessed for expression of MUC16 (mAb X75) and a PE-conjugated secondary Ab (red line). The secondary Ab alone served to establish the background fluorescence (black line). **B**, OVCAR3 cells in suspension were incubated with HEK293T-derived culture supernatant containing soluble mesothelin. Mesothelin binding was detected via anti-FLAG antibody staining (mAb M2) and a FITC-conjugated secondary Ab (green line). Cells treated with culture medium alone served as negative control (black line). **C**, OVCAR3 cells in suspension were incubated with HEK293T-derived culture supernatant containing Meso-TR3. To prevent binding of Meso-TR3 via TR3/death receptor interaction, Meso-TR3 was complexed with soluble DR5-Fc. Meso-TR3 binding was detected via anti-FLAG antibody staining similar to **(B)** using mAb M2, followed by FITC-conjugated secondary Ab (green line). Cells treated with culture medium alone served as negative control (black line). **D**, OVCAR3 cells were grown on 4-chamber slides and incubated the following day with Meso-TR3 complexed with DR5-Fc, similar to what has been described for **(C)**. After washing, the cells were stained with a mixture of MUC16 pAb (red) and FLAG mAb (green), respectively. The cells were counterstained with TOPRO3 (blue, nuclei) and analyzed by confocal microscopy. The individual channels were overlaid to document co-localization of tumor marker and the targeted cancer drug (Merge). Original magnification: 63 × .

As our primary model system for binding and functional killing experiments, we chose the well-characterized ovarian cancer cell line OVCAR3. These cells have been described to express MUC16 on their plasma membrane [21]. In addition, they are moderately sensitive to recombinant TRAIL [22-24]. In order to confirm the MUC16 expression profile on OVCAR3 cells, we performed flow cytometry and were able to detect a strong surface expression with a homogenous staining pattern for 100% of the cells (Figure 2A). We then tested the ability of soluble, FLAG-tagged recombinant mesothelin to bind to native, membrane-bound MUC16 employing an in vitro binding assay using OVCAR3 cells. We could indeed confirm that soluble mesothelin was capable of binding to OVCAR3 cells by flow cytometry (Figure 2B). The staining pattern correlated well with the MUC16 expression profile of this cell line as nearly 100% of the cells were positive for the FLAG epitope tag, i.e. bound recombinant mesothelin. This pilot experiment was crucial as it confirmed not only the binding capacity of recombinant mesothelin to native MUC16 but also demonstrated accessibility of the epitope tag in the context of the mesothelin/MUC16 interaction. To demonstrate broader applicability of our targeting concept, a similar study was also performed with the cervical cancer cell line HeLa (see below).

It was predicted that the multi-domain Meso-TR3 fusion protein would bind to OVCAR3 cells via two discrete mechanisms: 1) via the mesothelin/MUC16 interaction and 2) via the TR3/death receptor interaction (both DR4 and DR5 are expressed in OVCAR3 cells, data not shown and [24]). Since these circumstances would have complicated the interpretation of binding studies mediated exclusively via the mesothelin moiety of Meso-TR3, we first saturated the death receptor binding sites of Meso-TR3 with soluble death receptor 5 (DR5-Fc). In a following step, the Meso-TR3/DR5-Fc complexes were added to OVCAR3 cells in suspension. After several washing steps, the cells were stained for the presence of the FLAG epitope tag as evidence for drug binding to the OVCAR3 reporter cells. Using flow cytometry, we detected a strong and homogeneous fluorescence signal for cell-bound Meso-TR3, which was nearly identical to the MUC16 staining profile and similar to the binding pattern of soluble mesothelin alone (Figure 2C). Further evidence that Meso-TR3 and MUC16 co-localize on the plasma membrane of the target cells was obtained by employing confocal microscopy. Using the same detection system (anti-FLAG antibody) and death receptor blocking strategy (DR5-Fc pretreatment), we detected strong fluorescence signals for both the MUC16 eptiope (red) and the FLAG tag of Meso-TR3 (green) (Figure 2D), with no “cross-bleeding” between the detection channels (not shown). Importantly, the two signals overlapped (Figure 2D, “merge”) and suggest that Meso-TR3 co-localizes with the mesothelin receptor MUC16 on the same cancer cell membrane.

### The mesothelin/MUC16 interaction converts Meso-TR3 into a potent cancer drug

**Figure 3:**
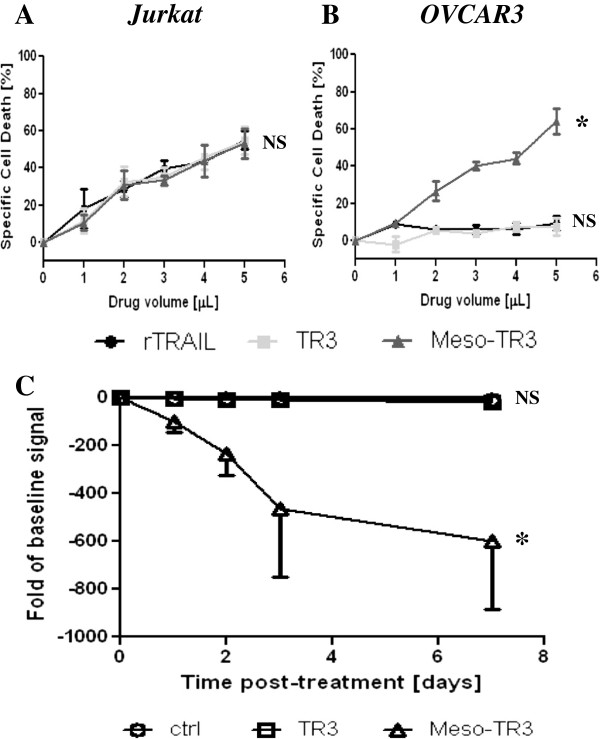
**Meso-TR3 is a targeted therapeutic on MUC16-expressing tumor cells. A**, The cell killing profiles of TR3, Meso-TR3 and rTRAIL [0.2 ng/μL] were established on the MUC16-deficient T cell leukemia cell line Jurkat. NS, not significant (one-way ANOVA). **B**, The same killing assay as in **(A)** using identical drug concentrations but the MUC16-positive ovarian cancer cell line OVCAR3 instead. *, *P* < 0.0075; NS, not significant (one-way ANOVA). **C**, NOD/SCID mice were injected i.p. with 2 × 10^6^ luciferase labeled OVCAR3 cells. The following day the baseline signal intensity was determined by live animal imaging. Three groups of mice (n = 7) were treated i.p. for 7 days with 300 μL medium alone (ctrl.), TR3 and Meso-TR3. Treatment efficiency is plotted as fold change (mean ± SEM) of signal intensity relative to the baseline signal obtained prior to treatment (negative data reflect a reduction in tumor burden). *, *P* < 0.016; NS, not significant (one-way ANOVA).

In order to compare the relative ability of cell death induction between Meso-TR3 and non-targeted TR3, it was important to establish the killing capacity of each drug mediated exclusively by the TR3 effector domain. Thus, we selected the TRAIL-sensitive leukemia cell line Jurkat which lacks expression of MUC16 (not shown). Under the assumption that the mesothelin epitope of Meso-TR3 would not impact the killing capacity of the MUC16-targeted TRAIL fusion protein, we established the killing curves for TR3, Meso-TR3 and recombinant TRAIL (rTRAIL) and identified conditions under which all TRAIL drugs induced cell death to the same degree in the absence of the tumor marker MUC16 (Figure 3A). This killing profile changed significantly when the same conditions were used to treat MUC16-positive OVCAR3 cells, known to be sensitive to recombinant TRAIL [22-24]. Non-targeted TR3 turned out to be quite inefficient with only ≈ 10% cell killing capacity at the highest dose used (Figure 3B). Importantly, TR3’s killing profile was identical to that of rTRAIL, which is consistent with our earlier findings in that both drugs activated the extrinsic death pathway equally well and suggested that each trimer assumes the same native conformation [12]. Treatment with Meso-TR3, however, resulted in an enhanced killing profile approaching 65% cell death at the highest drug dose employed in this experiment (Figure 3B). Linear regression analysis of the killing curves suggested a 7 to 12-fold stronger activity profile of Meso-TR3 when compared to TR3 and rTRAIL in OVCAR3 cells.

In order to obtain quantitative drug concentration data, we employed semi-quantitative Western blot an analysis. This detection method was selected as it does not rely on native protein conformations such as ELISA assays, especially because we began to suspect that the mesothelin domain of our Meso-TR3 fusion protein might have conformational properties which would interfere with the more commonly used ELISA-based assays. When drug concentrations which had achieved identical killing capacities on MUC16-negative Jurkat cells were compared, we consistently found ≈ 6 to 8-fold stronger signal intensities for Meso-TR3 compared to TR3 (Additional file 1: Figure S1). To address this difference in drug concentration, we increased the TR3 dose to that of Meso-TR3 and repeated the killing experiments with Jurkat and OVCAR3 cells. TR3 was now capable of killing more Jurkat cells than Meso-TR3 under these conditions (Additional file 2: Figure S2A). However, TR3’s impact on killing OVCAR3 cells did not improve, while Meso-TR3 was still more potent in killing MUC16 expressing OVCAR3 cells (Additional file 2: Figure S2B). Overall, these data show that a significantly higher concentration of Meso-TR3 is required to achieve equivalent biological effects on MUC16-deficient cells and, at equipotent doses, Meso-TR3 is substantially more effective than TR3 on MUC16-positive cancer cells.

In a next step, the killing activity of Meso-TR3 on MUC16-positive cancer cells was assessed in a preclinical mouse model of ovarian cancer. Luciferase-tagged OVCAR3 cells were injected via the intraperitoneal route into NOD/SCID mice. Luciferase activity was determined via non-invasive bioluminescence imaging [18]. The mice were treated via i.p. injections with medium only (control), or equipotent doses of either non-targeted TR3 or Meso-TR3 for 7 days (compare Figure 3A). The abdominal tumor burden (signal intensity) was then recorded at selected time points. The mice treated with Meso-TR3 responded with a robust decline in tumor burden by nearly a 600-fold reduction of the luciferase signal at 7 days post-treatment, while the control group and the mice treated with TR3 alone did not respond to the treatment (Figure 3C). These results suggest that native MUC16 is also available *in vivo* to anchor Meso-TR3 to the tumor cell membrane and that this tumor homing capacity directly corresponds with an enhanced target cell killing mechanism, in agreement with our in vitro killing data.

### The enhanced Meso-TR3-mediated cell death is due to its conversion into a membrane anchored TRAIL drug

**Figure 4:**
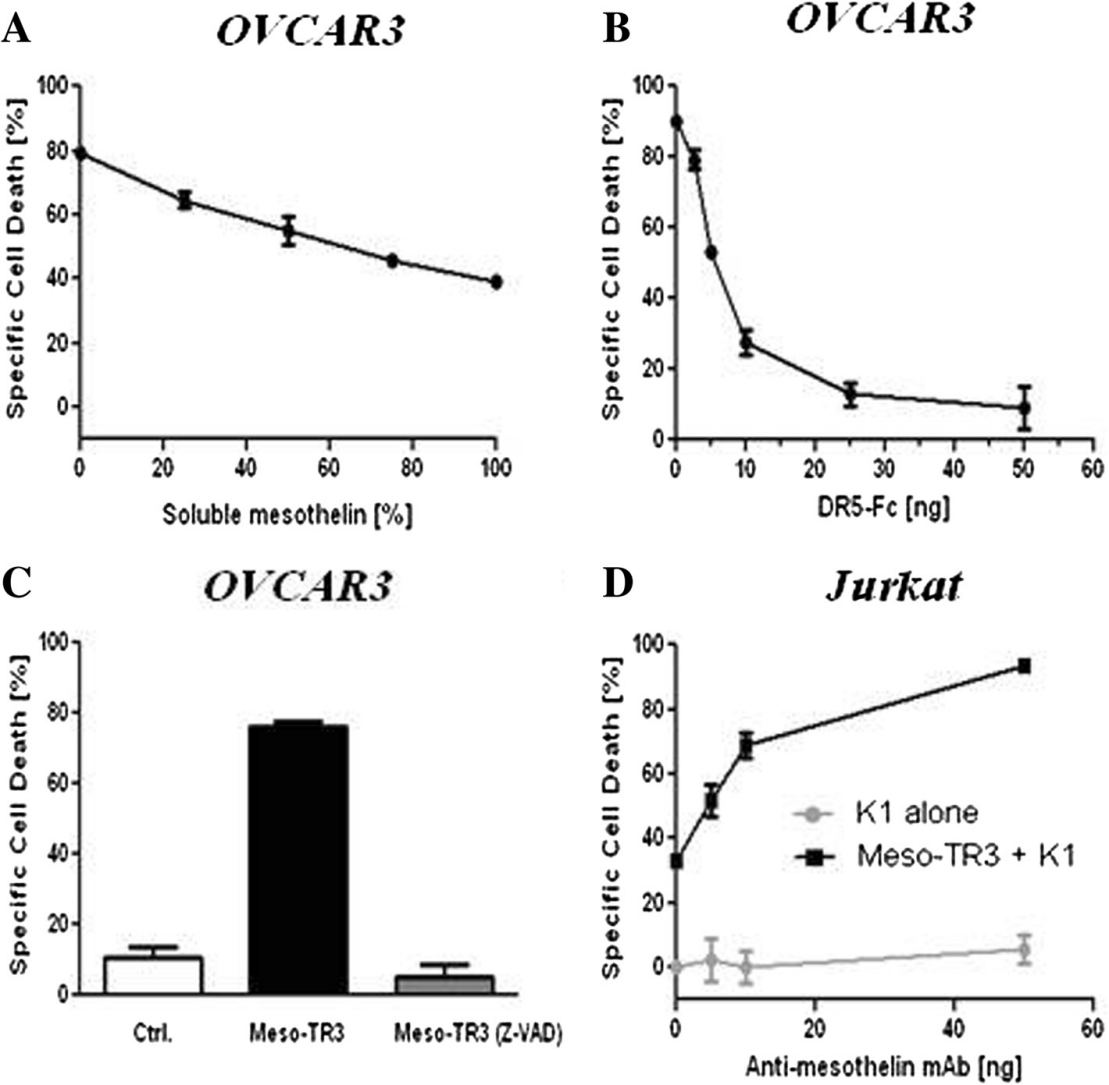
**Phenotypic characterization of MUC16-targeted Meso-TR3. A**, OVCAR3 cells were challenged with a constant amount of Meso-TR3 (80% specific cell death) and increasing concentrations of soluble mesothelin to study the impact of the mesothelin/MUC16 interaction of Meso-TR3. **B**, OVCAR3 cells were challenged with a constant amount of Meso-TR3 (90% specific cell death) and increasing concentrations of DR5-Fc to verify involvement of the extrinsic death pathway as a mechanism of Meso-TR3 killing. **C**, OVCAR3 cells were treated with a constant amount of Meso-TR3 (75% specific cell death) in the presence of Z-VAD-FMK, a pan-caspase inhibitor, vital mediators of apoptosis. Cells treated with DMSO were used as a control. *P* < 0.001 (one-way ANOVA). **D**, MUC16-deficient Jurkat cells were treated with low dose Meso-TR3 (33% specific cell death) in the presence of anti-mesothelin mAb. Cells treated with anti-mesothelin Ab alone served as a control. *P* < 0.0019 (Student’s t-test). Error bars, mean ± SD. Results are representatives of at least 2 independent experiments done in triplicates.

Based on the much enhanced killing profile of Meso-TR3 on MUC16-positive OVCAR3 cells, we hypothesized that the mesothelin/MUC16 interaction, i.e. the surface tethering of Meso-TR3 was responsible for the observed effects. To investigate this assumption, we performed a killing assay in the presence of increasing concentrations of soluble mesothelin to block the MUC16/Meso-TR3 interaction. As predicted, we were able to achieve a dose-dependent reduction in cell killing from 80% (no competitor) to 40% (highest competitor dose) (Figure 4A). We did not expect complete protection from apoptosis of cells treated with Meso-TR3, even assuming 100% MUC16 blockade with soluble mesothelin, since all TRAIL variants (including TR3, recombinant rTRAIL and Meso-TR3) exhibit baseline apoptosis-inducing activities in MUC16-deficent cancer cells due to direct interaction of the TRAIL timer with cell surface DR4/5.

In order to rule out phenotypic alterations that the addition of the MUC16 targeting moiety mesothelin to the TR3 drug platform might have caused, we asked if the induction of cell death was exclusively mediated via the extrinsic death receptor pathway. Two lines of evidence suggest that this mechanism is well preserved following Meso-TR3 treatment. First, when soluble DR5-Fc was added to a standard killing assay using MUC16-positive OVCAR3 cells, Meso-TR3’s killing capacity was nearly completely blunted, evidenced by a gradual decrease in cell death from 90% in the absence of the soluble receptor to below 10% at the highest DR5-Fc concentration (Figure 4B). Further evidence for the involvement of the death receptor signaling cascade induced by Meso-TR3 was obtained employing the pan-caspase inhibitor Z-VAD-FMK, which blocks intracellular caspases activated via the extrinsic death pathway of apoptosis. Z-VAD-FMK turned out to protect the cells completely from Meso-TR3-induced cell death (Figure 4C).

Higher order TRAIL aggregates have been associated with increased activity due to more efficient death receptor clustering. This clustering, especially regarding the DR5 receptor, is not exclusively cancer cell selective and is generally considered undesired due to unintentional side effects exerted on normal host cells also capable of expressing DR4 and/or DR5 [25]. In an attempt to test if Meso-TR3 forms higher order aggregates in solution, we treated Jurkat cells with Meso-TR3 in the presence of a monoclonal antibody (K1), directed against the mesothelin moiety of the fusion protein. Using a sublethal dose of Meso-TR3 (33% cell death), we were able to demonstrate a dose-dependent augmentation of cell death to nearly 100% at the highest concentration of cross-linking mesothelin antibody (Figure 4D). These results strongly suggest that Meso-TR3 does indeed assume a monomeric configuration in solution that can be functionally enhanced by forming higher order aggregates (Meso-TR3 dimers). In contrast to earlier toxicity concerns of utilizing aggregated TRAIL preparations for clinical applications, Meso-TR3 would only form higher order clusters at the tumor cell membrane following interaction with multiple binding sites on MUC16, which then converts the soluble drug into a highly potent membrane anchored tumor cell killer.

### Meso-TR3 kills in a MUC16-selective manner

**Figure 5:**
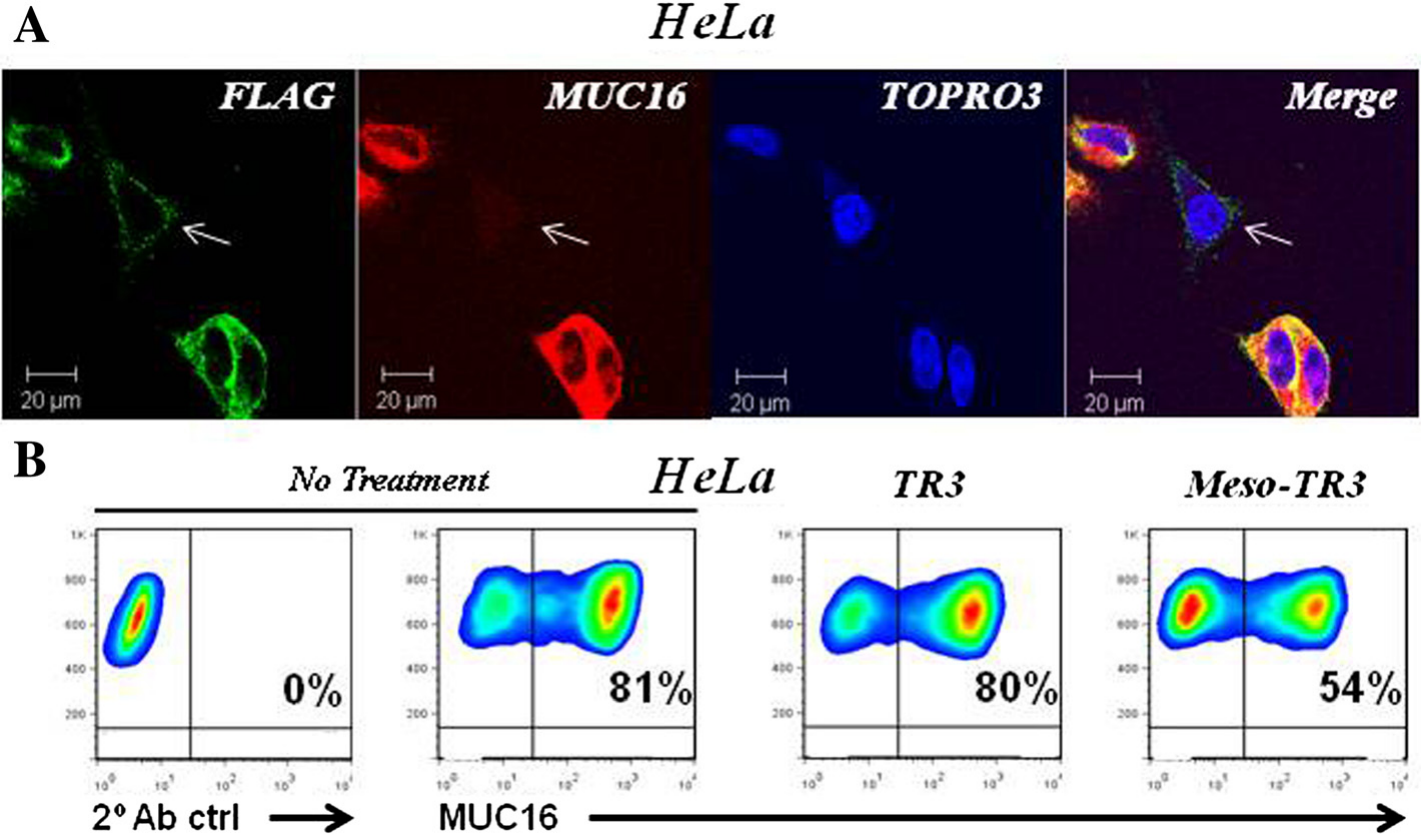
**Meso-TR3 preferentially kills MUC16-expressing tumor cells. A**, HeLa cells were grown on 4-chamber slides and incubated the following day with Meso-TR3 complexed with DR5-Fc. After washing, the cells were stained with a mixture of MUC16 pAb (red) and FLAG mAb (green), respectively. The cells were counterstained with TOPRO3 (blue, nuclei) and analyzed by confocal microscopy. The individual channels were overlaid to document co-localization of tumor marker and the targeted cancer drug (Merge). Original magnification: 63×. **B**, HeLa cells were treated with TR3 and Meso-TR3 for 24 h. Two days post-treatment, the cells were assessed for changes in the MUC16 ratio using flow cytometry. Representative density plots are shown from experiments done at least twice in duplicates.

In order to study target selectivity aspects of Meso-TR3 toward MUC16-expressing cancers, we took advantage of the fact that the cervical cancer cell line HeLa is comprised of a native mix of MUC16-positive and negative cells (80% and 20%, respectively). We therefore performed confocal microscopy on HeLa cells to study the surface tethering of Meso-TR3 using the same conditions described above for drug binding to OVCAR3 cells (Figure 2D). We found that cells positive for the MUC16 tumor marker were heavily coated with Meso-TR3 (Figure 5A), while cells with nearly undetectable antigen expression were incapable of capturing Meso-TR3 and stained only weakly for the targeted drug (Figure 5A, arrow). Based on these findings, we anticipated that Meso-TR3 would have a higher affinity for the MUC16-positive population and selectively eliminate these from the HeLa cell pool. Under conditions where both drugs killed the same number of MUC16-negative Jurkat cells, HeLa cells were more susceptible to Meso-TR3 (Additional file 3: Figure S3), similar to what we have shown for OVCAR3 cells. In addition to its much enhanced killing profile on HeLa cells, Meso-TR3 treatment resulted in a more than 30% reduction of the MUC16-positive cell population from 80% to 54% (Figure 5B). In contrast, non-targeted TR3 was incapable of shifting the MUC16 ratio in this cervical cancer cell line due to its non-targeted nature. Along these lines, and to further underscore the universal applicability of our targeted drug delivery concept, similar results were obtained with BxPC3 pancreatic cancer cells in vitro (data not shown).

## Discussion

If we were to seek an ideal cancer therapeutic, we would design a drug that could be delivered systemically, seek out its target autonomously, ignore all non-targets and, upon arrival at its destination, would fully unleash its intended pharmacologic activity. Such a selective activity profile might be most beneficial for the treatment of human malignancies wherein treatment with conventional chemotherapy is known to be associated with debilitating side effects directly linked to an adverse impact on the quality of life of the patients.

Nearly 20 years ago, the TNF superfamily member TRAIL was identified and it immediately became apparent that it exhibited many properties of an ideal cancer therapeutic because of its strong apoptosis induction on transformed cancer cells and lack of harmful side effects for the host. Since then, TRAIL has been evaluated in a number of clinical trials and found to be effective against several types of cancers [26]. In order to make improvements in the field of TRAIL-based drug development, investigators have looked for ways to stabilize the bioactive trimer by several means, including incorporation of trimerization domains or by simply adding Zn^2+^ to the production process which aids the coordination of the three cysteines present once in each TRAIL domain [27]. The next step toward more versatile TRAIL drugs was achieved by incorporating targeting moieties directed against cancer-specific surface markers. Cancer targeting was primarily achieved using antibody fragments (scFv) on the basis of the conventional monomeric TRAIL cDNA [11,28]. This technology turned out to be quite effective, despite a 1:1 stoichiometry of the targeting and effector domain of the fusion proteins which could potentially interfere with the formation of bioactive TRAIL trimers. In fact, we have produced scFv-TRAIL fusion proteins in mammalian cells employing two different antibody fragments with one drug being constitutively active (regardless of the presence of a secondary cancer antigen), while the other drug was only active in the presence of the target antigen (D. Spitzer, unpublished data). These results underscore the unpredictability of drug properties that could result from exchanging targeting moieties (scFv) generally accepted to have nearly identical three dimensional structures.

We have recently designed a new method to produce bioactive soluble TRAIL from mammalian cells, designated TR3, not feasible when attempted in the context of a monomeric expression format [29]. In addition to its much enhanced stability compared to certain recombinant TRAIL preparations [12], this genetically fused TRAIL trimer has the capacity to serve as a drug platform for the design of targeted cancer therapy under stoichiometric control. For example, fusing a scFv to the N-terminus of TR3 resulted in a RBC-targeted scFv-TR3 fusion protein with a favorable 1:3 stoichiometry that was capable of tethering human TR3 to mouse RBCs which were thereby converted into potent effector surfaces in analogy to nanoparticles, capable of facilitating robust bystander (trans) cell killing [12]. In our current study, we characterized a tumor-targeted variant of TR3 by harnessing the strong binding affinity of the two well described biomarkers mesothelin and MUC16. MUC16 is not only an established biomarker in ovarian cancer, it has also been explored as a promising target for cancer therapy. In a recently reported phase I clinical trial of DMU-C5754A (an anti-MUC16 antibody/mitotic toxin MMAE drug conjugate), objective responses were observed in 5 out of 44 patients diagnosed with advanced/recurrent platinum-resistant ovarian cancer [30]. All five patients who responded to the conjugate had high expression levels of MUC16 which underscores the importance of targeting strategies in increasing the effectiveness of cancer drugs by way of facilitating their efficient delivery to the tumor cells. Instead of targeting TR3 to MUC16 via an antibody-based strategy, we generated Meso-TR3, in which the mature form of human mesothelin was placed at the N-terminus of human TR3. Meso-TR3 bound avidly to endogenous MUC16, identical to soluble mesothelin itself and triggered a robust death pathway that involved the same signaling pathways as conventional TRAIL does (blocked by soluble DR5-Fc and global caspase inhibition with Z-VAD-FMK). These results have important implications because they confirm that the mesothelin targeting moiety is not masked by TR3 as it is still accessible to interact with membrane-associated MUC16 in vitro. Of note, the target recognition property of Meso-TR3 was retained in a metastatic xenograft mouse model of ovarian cancer under physiologic conditions in vivo.

Our results indicate that addition of mesothelin to the TR3 domain as in Meso-TR3, increases the effectiveness of TR3 in MUC16-positive malignancies such as ovarian and cervical cancers (shown for OVCAR3 and HeLa cell lines), while limiting its bioactivity on MUC16-negative Jurkat cells. These findings have important clinical implications with regard to maximizing the on-target effect of TRAIL while keeping its off-target effects to a minimum. The enhanced bioactivity of Meso-TR3 likely results from the enrichment of the cancer drug at the tumor cell membrane. This mesothelin/MUC16 ligand/receptor interaction serves to anchor soluble TRAIL to the surface of MUC16-positive cancer cells, thus converting the soluble drug into a membrane bound form of TR3. This conversion has been proposed to lead to a more efficient receptor clustering (particularly important for DR5-induced apoptosis), which provides a more potent death signal resulting in enhanced apoptosis compared to its soluble counterpart [31]. The importance of TRAIL receptor clustering in cell death is further exemplified by an enhanced induction of apoptosis noted in our experimental system upon adding mesothelin antibody to dimerize Meso-TR3 in solution, a concept that has just recently being explored to treat highly vascularized cancers [32]. Another important aspect of the mesothelin/MUC16 interaction is its contribution to both homotypic (tumor cell-tumor cell) and heterotypic (tumor cell-mesothelial cell) cell interactions [33]. The latter type of interaction is believed to promote adherence of tumor cells to the peritoneum, resulting in metastatic spread of the primary lesion into the abdomen [13,34,35]. These considerations suggest that by virtue of binding to MUC16, Meso-TR3 may also saturate and therefore reduce/eliminate the available binding sites on MUC16 for adhesive interactions with mesothelin-expressing normal endothelium, thus limiting the peritoneal dissemination of tumor cells in addition to augmenting TRAIL-mediated target cell death [36].

**Figure 6:**
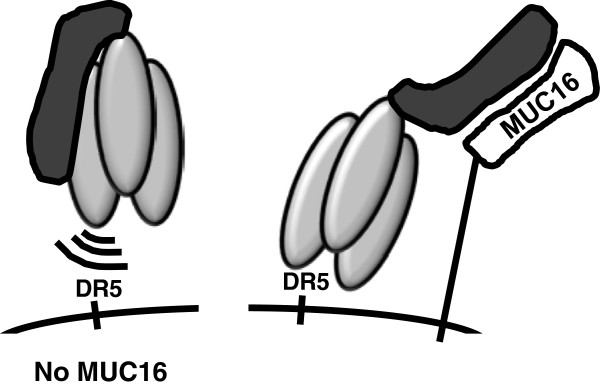
**Proposed mechanism of Meso-TR3’s pro-drug properties.** The mesothelin moiety of Meso-TR3 partially interferes with an unrestricted interaction of the TR3 domain and its death receptors (left panel). In the presence of MUC16 on the cancer cell surface, the mesothelin targeting domain is removed from the TR3 surface thus enabling unrestricted access to and full activation of the death receptor-mediated extrinsic death pathway (right panel).

Based on semi-quantitative Western blot analyses, an ≈ 6 to 8-fold higher concentration of Meso-TR3 was required to achieve the same biological effect as untargeted TR3 on MUC16-deficient Jurkat cells. This finding was unexpected since we reported earlier that we had not observed detrimental effects on the killing activity of a variety of domain additions engineered onto the TR3 drug platform [12]. A possible explanation for this finding is that in its native state, the steric relationship between mesothelin and TR3 in the context of the Meso-TR3 fusion protein might partially mask the DR/TR3 interaction leading to a reduced killing activity on MUC16-deficient cells (Figure 6, left panel). Only when the mesothelin targeting moiety binds to MUC16, full exposure of the TR3 trimer is enabled and results in an unrestricted accessibility with the surface-associated death receptor(s) DR4/5. We therefore propose that these structural changes, in combination with a now membrane tethered TR3 are responsible for Meso-TR3 to acquire its full cytotoxic potential at the target cell membrane (Figure 6, right panel).

## Conclusions

We have described the characterization of a downstream modification of the novel TRAIL-based drug platform TR3 which has many highly desirable anticancer properties. Soluble Meso-TR3 targets the cancer biomarker MUC16 *in vitro* and *in vivo* and exhibits all the positive features of a traditional TRAIL-based cancer drug, as well as enhanced stability, enhanced killing capacity and favorable 1:3 stoichiometry of targeting (mesothelin) and effector domain (TR3). Since TR3 and its targeted variants engage the same mechanistic pathways that have been explored for nearly 20 years with rTRAIL, we expect that Meso-TR3 activity could be further enhanced in combination with chemo and/or radiation therapy [37-39]. Importantly, the prodrug feature of Meso-TR3 should make it less toxic to normal cells while enhancing the effects on its cancer targets. We are currently in the process of expanding our preclinical xenograft models to primary, MUC16-expressing human malignancies including ovarian and pancreatic cancers. If efficacy and reduced toxicity in combination with chemotherapy can be confirmed in animal models, we anticipate moving Meso-TR3 into the clinical arena.

## Abbreviations

DR: Death receptor; DR5-Fc: Soluble death receptor 5-human Ig Fc fusion protein; Meso-TR3: Mesothelin-TR3 fusion protein targeting the tumor marker MUC16 (CA125); rTRAIL: Commercially available recombinant TRAIL; TNF: Tumor necrosis factor; TR3: Genetic fusion of three consecutive monomeric TRAIL domains; TRAIL: TNF-related apoptosis-inducing ligand.

## Competing interest

Dirk Spitzer and William Hawkins own patent rights for the TR3 drug platform. All other authors declare no conflict of interest.

## Authors’ contributions

GG performed the experiments, analyzed the data and wrote the manuscript. JG was involved in cloning TR3 expression constructs, drug production and performed in vivo drug testing. BB performed confocal microscopy. MAP, DGM and PG were involved in critical review of the manuscript. LC and DPW were involved in BLI imaging and data analyses. WGH was involved in critical review of the manuscript. DS designed the TR3 expression constructs, designed the experiments, analyzed the data and reviewed the manuscript. All authors have read and approve the final version of the manuscript.

## Pre-publication history

The pre-publication history for this paper can be accessed here:

http://www.biomedcentral.com/1471-2407/14/35/prepub

## Supplementary Material

Additional file 1: Figure S1 Drug quantification via Western blot analysis. TR3 and Meso-TR3 preparations exerting identical killing profiles on MUC16-deficient tumor cells (compare Figure 3**A**) were subjected to semi-quantitative Western blot analysis under reducing conditions using anti-TRAIL pAb. The immunoreactive bands were quantified using QuantityOne software on a BioRad imaging system, with Meso-TR3 approximately 6 to 8-fold more abundant than TR3. Click here for file

Additional file 2: Figure S2 Meso-TR3 enhances tumor cell killing on MUC16-positive ovarian cancer cells. Based on the ≈ 6 to 8-fold lower TR3 signal intensity on Western blot analysis (Additional file 1: Figure S1), the TR3 concentration was increased 6-fold to match that of Meso-TR3. **A**, The cell killing profiles of TR3 and Meso-TR3 were established on the MUC16-deficient T cell leukemia cell line Jurkat. **B**, The same conditions were applied to the MUC16-positive cell line OVCAR3. Statistical analysis was calculated using the Student’s t-test (mean ± SEM). Click here for file

Additional file 3: Figure S3 Meso-TR3 has increased bioactivity on MUC16-positive cervical cancer cells. **A**, The cell killing profiles of TR3 and Meso-TR3 were established on the MUC16-deficient T cell leukemia cell line Jurkat as described in Figure 3**A**, with an ≈ 6 to 8-fold lower TR3 signal intensity on Western blot analysis (Additional file 1: Figure S1). **B**, The same conditions were then applied to the MUC16-positive cervical cancer cell line HeLa. Due to a more rapid cell death induction of Meso-TR3 in this cell line, the killing assay for both cell lines was initiated 6 h post-treatment. Statistical analysis was calculated using the Student’s t-test (mean ± SEM). Click here for file
