# Supplementary figures and images for: Novel treatment option for MUC16-positive malignancies with the targeted TRAIL-based fusion protein Meso-TR3
Source: BMC Cancer. 2014 Jan 21;14:35. doi: 10.1186/1471-2407-14-35 (PMC3903436; doi:10.1186/1471-2407-14-35)

Additional Fig. S1

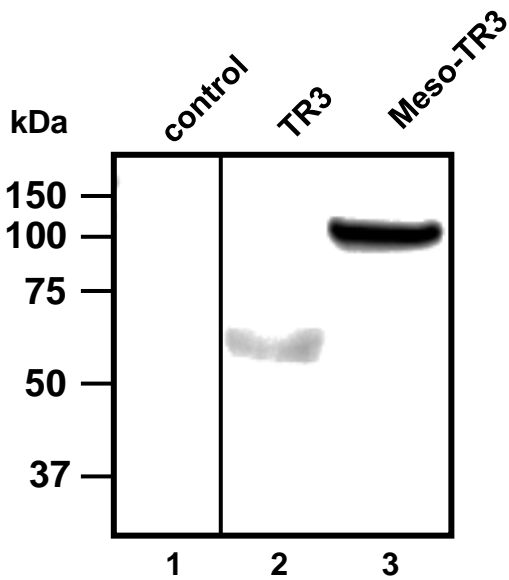

Supplement: Additional file 1: Figure S1 — Drug quantification via Western blot analysis. TR3 and Meso-TR3 preparations exerting identical killing profiles on MUC16-deficient tumor cells (compare Figure 3A) were subjected to semi-quantitative Western blot analysis under reducing conditions using anti-TRAIL pAb. The immunoreactive bands were quantified using QuantityOne software on a BioRad imaging system, with Meso-TR3 approximately 6 to 8-fold more abundant than TR3. [file 1471-2407-14-35-S1.pdf]

**A**

*Jurkat*

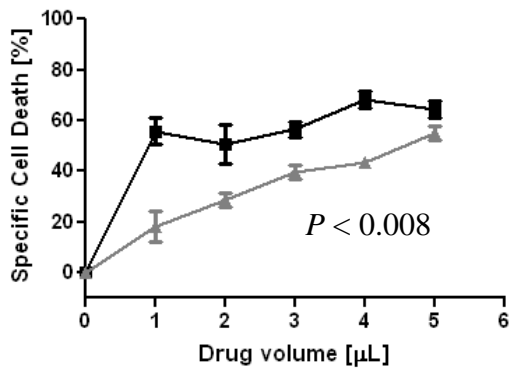

■ TR3

**B**

*OVCAR3*

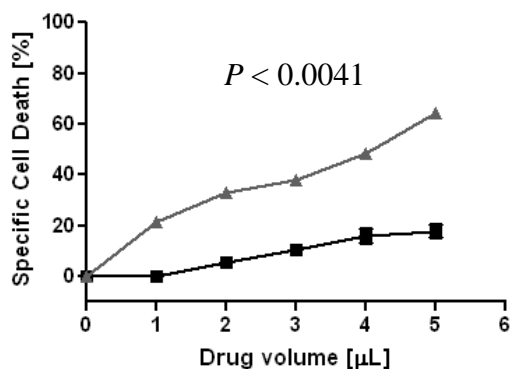

▲ Meso-TR3

Supplement: Additional file 2: Figure S2 — Meso-TR3 enhances tumor cell killing on MUC16-positive ovarian cancer cells. Based on the ≈ 6 to 8-fold lower TR3 signal intensity on Western blot analysis (Additional file 1: Figure S1), the TR3 concentration was increased 6-fold to match that of Meso-TR3. A, The cell killing profiles of TR3 and Meso-TR3 were established on the MUC16-deficient T cell leukemia cell line Jurkat. B, The same conditions were applied to the MUC16-positive cell line OVCAR3. Statistical analysis was calculated using the Student’s t-test (mean ± SEM). [file 1471-2407-14-35-S2.pdf]

**A** *Jurkat*

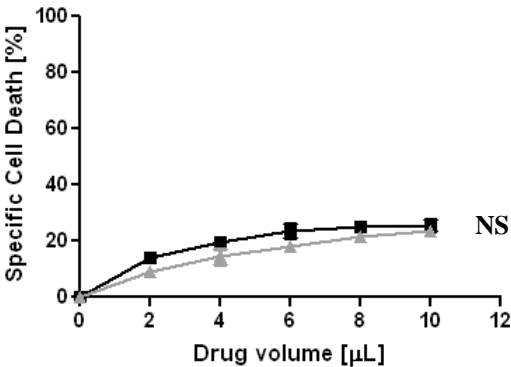

■ TR3    ▲ Meso-TR3

**B** *HeLa*

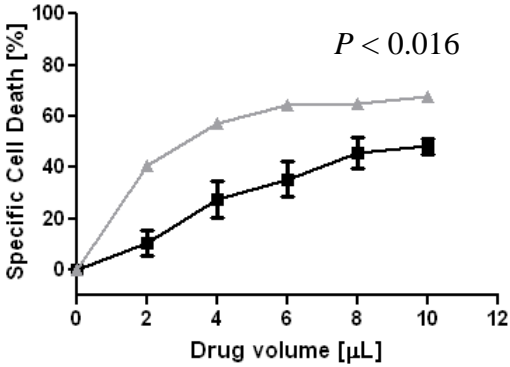

Supplement: Additional file 3: Figure S3 — Meso-TR3 has increased bioactivity on MUC16-positive cervical cancer cells. A, The cell killing profiles of TR3 and Meso-TR3 were established on the MUC16-deficient T cell leukemia cell line Jurkat as described in Figure 3A, with an ≈ 6 to 8-fold lower TR3 signal intensity on Western blot analysis (Additional file 1: Figure S1). B, The same conditions were then applied to the MUC16-positive cervical cancer cell line HeLa. Due to a more rapid cell death induction of Meso-TR3 in this cell line, the killing assay for both cell lines was initiated 6 h post-treatment. Statistical analysis was calculated using the Student’s t-test (mean ± SEM). [file 1471-2407-14-35-S3.pdf]
